# Extended reality training for mass casualty incidents: a systematic review on effectiveness and experience of medical first responders

**DOI:** 10.1186/s12245-024-00685-3

**Published:** 2024-08-23

**Authors:** María del Carmen Cardós-Alonso, Lucía Otero-Varela, María Redondo, Miriam Uzuriaga, Myriam González, Tatiana Vazquez, Alberto Blanco, Salvador Espinosa, Ana María Cintora-Sanz

**Affiliations:** 1 Servicio de Urgencias Médicas de la Comunidad de Madrid (SUMMA112), Madrid, Spain; 2 Departamento de Enfermería, Universidad Complutense de Madrid, Madrid, Spain; 3 Fundación para la Innovación e Investigación Biosanitarias en Atención Primaria (FIIBAP), Madrid, Spain; 4 Fundación Española de Reumatología (FER), Madrid, Spain

**Keywords:** Disaster preparedness, Emergency medicine education, Disaster training, Extended reality

## Abstract

**Introduction:**

Mass casualty incidents (MCI) are unforeseeable and complex events that occur worldwide, therefore enhancing the training that medical first responders (MFRs) receive is fundamental to strengthening disaster preparedness and response. In recent years, extended reality (XR) technology has been introduced as a new approach and promising teaching technique for disaster medicine education.

**Objective:**

To assess the effectiveness of XR simulation as a tool to train MFRs in MCIs, and to explore the perception and experience of participants to these new forms of training.

**Design:**

Systematic review.

**Methods:**

This systematic review was conducted in accordance with the “Preferred reporting items for systematic reviews and meta-analyses” (PRISMA) statement. Four databases were searched (MEDLINE, EMBASE, CINAHL and LILACs) using a comprehensive search strategy to identify relevant articles, and MetaQAT was used as a study quality assessment tool. Data from included studies was not pooled for meta-analysis due to heterogeneity. Extracted data was synthesised in a narrative, semi-quantitative manner.

**Results:**

A total of 18 studies were included from 8 different countries. Studies encompassed a variety of participants (e.g., nurses, paramedics, physicians), interventions (virtual, mixed and augmented reality), comparators (comparison between two groups and single groups with pre-post evaluation), and outcomes (effectiveness and MFR perception). The synthesis of data indicated that XR was an effective tool for prehospital MCI training by means of improved triage accuracy, triage time, treatment accuracy, performance correctness and/or knowledge acquired. These XR systems were well perceived by MFRs, who expressed their interest and satisfaction towards this learning experience and emphasized its usefulness and relevance.

**Conclusion:**

This research supports the usefulness and significance of XR technology that allows users to enhance their skills and confidence when facing forthcoming disasters. The findings summarize recommendations and suggestions for the implementation, upgrade and/or assessment of this novel and valuable teaching method.

**Supplementary Information:**

The online version contains supplementary material available at 10.1186/s12245-024-00685-3.

## Introduction

Disasters and mass casualty incidents (MCI), either man-made or natural, are unforeseeable events that occur worldwide. In recent years, there has been a shift in focus from post-disaster improvisation to pre-disaster preparedness, which is crucial to minimising the impact of disasters on communities and to save lives [1]. The League of Red Cross Societies and the United Nations Disaster Relief Office work together to promote pre-disaster planning in vulnerable countries. This involves coordinating efforts and sharing information among agencies involved in disaster relief. In addition, the need for professional disaster management expertise is increasingly recognised. A number of groups and organisations have been established to provide expertise and support in this field. Private initiatives, meetings, research centres and projects have also been launched to improve disaster preparedness and response. Overall, the increase in awareness and action towards pre-disaster preparedness indicates a significant change in the mindset and approach to managing and mitigating the impact of disasters effectively [2].

MCIs are complex by nature, with significant variability due to the type of incident, number of victims, availability of resources, timing, weather conditions, or even the triage system used [3]. In consequence, developing a curriculum to teach emergency personnel how to manage victims appropriately in this environment is inherently challenging [4] because of the difficulty of recreating these scenarios. Up to recently, training has been carried out using tabletop exercises and large-scale simulation drills where a large number of human resources and equipment are needed. This makes it hazardous and expensive to repeat frequently, when actually, previous research indicates that higher training frequency and better training quality are associated with improved disaster preparedness [5]. Thus, it is understandable that many healthcare professionals, including medical first responders (MFRs), feel that their preparedness for disaster response is inadequate.

As more advanced pedagogical technologies are developed, teaching in MCIs and disasters is being upgraded. In recent years, there has been an increasing trend in the use of extended reality (XR) systems to support emergency and disaster risk management, with special emphasis on training [6]. XR encompasses virtual, augmented and mixed reality (VR, AR, and MR, respectively), and it has grown in popularity as it offers multiple advantages, such as the possibility to train safely without direct risk to participants and the opportunity for repetitive practice to acquire a certain level of competence and to improve retention [7–9]. Furthermore, some systems integrate realistic scenarios and immersive environments that allow trainees to increase the speed of gaining knowledge, and to develop high-quality skills [10, 11].

Enhancing the training MFRs receive is fundamental to strengthening the preparedness and response to MCIs and disasters. Other reviews have identified relevant articles highlighting what could be a promising and novel tool for disaster medicine, although the effectiveness of the training was not assessed [7, 10]. In addition, our project partners [12] evaluated the effectiveness of different disaster training programmes, including traditional techniques. Our study, however, focused on the use of emerging technologies (i.e., extended reality) as training methods, and we also aimed to explore the perception and experience of participants to these new forms of training, not limited to assessing their technical skills.

Consequently, a systematic review of the literature was performed, following the Preferred Reporting Items for Systematic Reviews and Meta-Analyses guidelines [13], to address the following research questions:

1. Primary question: What is the effectiveness of XR simulation as a tool to train MFRs in mass casualty incidents?
2. Secondary question:
  What tools and metrics were used to measure the effectiveness of the XR simulation training?
  What is the experience of MFRs training for mass-casualty incidents using XR simulation? What is their perceived impact of such training?

## Methods

A detailed description of the methodology used is available in the published study protocol (PROSPERO #CRD42021275692) [14].

### Eligibility criteria

Detailed inclusion and exclusion criteria are outlined in the study protocol [14]. To conduct a thorough systematic review, all original research reporting on XR training for medical first responders, i.e., experimental, quasi-experimental, and observational studies included. The study population were MFRs, defined as healthcare professionals from the emergency medical service, including students and residents, but excluding in-hospital healthcare providers. The intervention included educational activities or training using extended reality simulation: virtual reality, mixed reality and augmented reality (Table 1). Studies with no control group (e.g., before and after) and those comparing groups of participants using different interventions (i.e., XR vs. another type of simulation or training) were selected. The settings were simulated scenarios of mass casualty incidents in an out-of-hospital environment, including those caused by natural disasters or human-made.

**Table 1:** Definition of interventions [6, 15], and outcomes

Intervention	Definition
Virtual reality simulation (VRS)	Screen-based VR that uses three-dimensional environments and desktop computer interface. VRS allows participants to interact within a virtual environment using normal computer accessories, such as mouse and keyboard controls. It is the least immersive of these technologies.
Virtual reality (VR)	In VR, users are fully immersed in a virtual environment with virtual elements that obscure the physical environment and simulate the physical objects. Using a head-mounted display or headset, users can experience a computer-generated world with images and sounds. They also can manipulate objects and move around using touch controllers while connected to a console or PC.
Augmented reality (AR)	AR superimposes digital information on real-world elements. AR scenes occur in the real world and incorporate non-dominating virtual objects that interact with the user and/or the real environment without completely obscuring it. Accordingly, AR keeps the real world at the centre but enhances it with other digital details, overlaying new layers of perception and complementing the reality or environment.
Mixed reality (MR)	MR brings together the natural world and digital elements, thus providing a means to work synchronously across physical and virtual domains. MR allows users to see and immerse themselves in the world, even when interacting with a virtual environment using state-of-the-art sensing and imaging technologies, without the need to remove their headsets. In addition, it provides the ability to have one foot (or type) in the real world and the other in an imaginary place, that is, breaking down the basics between the real and the imaginary.
**Outcome**	**Definition**
Triage accuracy	Skill of classifying each casualty into the appropriate triage category, comparing the ‘expected’ triage category with the category actually assigned by the participant.
Treatment/ Intervention accuracy	Accuracy of treatments assigned or lifesaving interventions conducted, focusing on individual decision-making rather than the ability to perform a task.
Time to triage	Time taken to complete each scenario or victim’s triage.
Knowledge acquired	The cognitive learning achieved.
Performance correctness	First-responder actions and decision-making, following the correct procedure. For instance, the ability to ensure the safety of the scene, call for additional resources and personnel, determine the status of victims in a timely fashion, place victims in the appropriate triage category, and apply a visible, correctly labelled triage tag to each victim during their simulated drills.
Participant perception/ experience	Satisfaction, opinion, acceptability, usability or self-efficacy.

The outcomes of interest are the effectiveness of the XR simulation training for MFRs in mass casualty incidents and the perception of MFRs. There is no standard definition for these outcomes and how to assess them; thus, these were determined based on the information reported in the included articles (Table 1). For instance, participants’ experience could be interpreted as satisfaction, opinion, acceptability, or usability. At the same time, effectiveness can also have many definitions, including triage accuracy, time to triage, or intervention correctness. Studies describing the development of an XR simulation but not reporting the training effectiveness were excluded.

These outcomes were classified within Kirkpatrick levels (Table 2), as it is the most effective and most used approach for evaluating training [16]. The metrics and tools used for effectiveness were also captured to better address the secondary questions.

**Table 2:** Kirkpatrick levels [16]

Levels	Definition
I - Reaction	A measure of how participants feel about the training program
II - Learning	An objective, quantifiable measure of how well trainees have acquired knowledge, improved skills, or changed attitudes due to training
III - Behaviour	A measure of how well behaviours learned in training is performed on the job (i.e., transfer of training)
IV - Outcome	A measure of how well training relates to final results, such as improved patient outcomes, reduced costs, enhanced quality

### Information sources and search strategy

The following databases were searched with no date, country, or language restrictions: MEDLINE (Medical Literature Analysis and Retrieval System Online), EMBASE (Excerpta Medica Database), CINAHL (Cumulative Index to Nursing and Allied Health Literature), and LILACS (*transl*. Latin American and Caribbean Literature in Health Sciences). To capture additional studies, grey literature was reviewed. Field experts were also contacted for further information about ongoing or unpublished studies.

After consulting with a research librarian, the search strategy was organized into four broad themes: [1] medical first responders (participants); [2] mass casualty incidents (setting); [3] education and simulation (intervention); and [4] extended reality (intervention). MeSH terms, titles and abstracts, and keywords were searched within each theme using the Boolean operator “OR”, and the four searches were then combined using the Boolean operator “AND”. The proposed keywords and MeSH terms used are available within the study protocol, as well as an example search strategy [14].

### Selection process and data collection

Both abstract and full-text screening phases will be done independently and in duplicate with the support of Covidence. This web-based software platform streamlines the screening and data extraction processes. Identified records were compiled in the reference management software Endnote™, and then uploaded into Covidence. Following the above eligibility criteria, titles and abstracts were first scanned to select articles for in-depth analysis if: (1) both reviewers agreed or (2) the abstract did not provide sufficient information to decide. During the full-text screening stage, studies were selected if agreement was found. Conflicts between reviewers were identified and discussed by the team until an agreement was reached. Study authors were contacted when necessary for further clarification if crucial information was missing from the included articles.

Two authors independently extracted relevant information about each included study: first author, publication year, country, study design, setting, number and type of participants, and details about the intervention, including type of XR, comparator, training content and duration. Results regarding the effectiveness of the training and how effectiveness was measured (i.e., tools and metrics) were collected and classified within Kirkpatrick levels. Participants’ experiences from the disaster training were also captured. The accuracy of the data extraction process was guaranteed by a third reviewer, who ensured consensus was achieved. Additionally, the data extraction form included the different items comprising the risk of the bias assessment tool.

### Risk of bias assessment

MetaQAT was used as a risk of bias and study quality assessment tool [17], given its versatility. As stated in the eligibility criteria, all original studies (experimental, quasi-experimental and observational) could be included; therefore, it was preferable to use one tool that allowed for evaluating individual studies regardless of their research design, like MetaQAT. Furthermore, assessing all studies with the same tool could help prevent possible biases and heterogeneity, as opposed to simultaneously using different tools that are specific to determined study designs (e.g., Cochrane risk-of-bias tool for RCTs).

MetaQAT is a validated tool that allows for rigorous appraisal, consisting of 8 items to assess each study’s relevancy, reliability, validity, and applicability [17]. As a result, the risk of bias was assessed at the study level.

### Data synthesis and analysis

Data from included studies was not pooled for meta-analysis due to the heterogeneity in the design, population, intervention and/or outcome. Consequently, results were presented in a narrative form with semi-quantitative analysis using descriptive statistics, thus, a qualitative synthesis was carried out.

Identified studies (both included and excluded) were summarised in a PRISMA flow diagram, and data extracted from those selected was tabulated into study characteristics and summary findings. Results from the included studies were elaborated in detail: relevant elements were reported and summarised for each type of XR simulation training. Further, to better address the research questions, study findings were stratified by the classification within Kirkpatrick’s evaluation model. The tools and metrics used to measure effectiveness were described. Participants’ experience was also outlined.

## Results

### Study characteristics

**Fig. 1:**
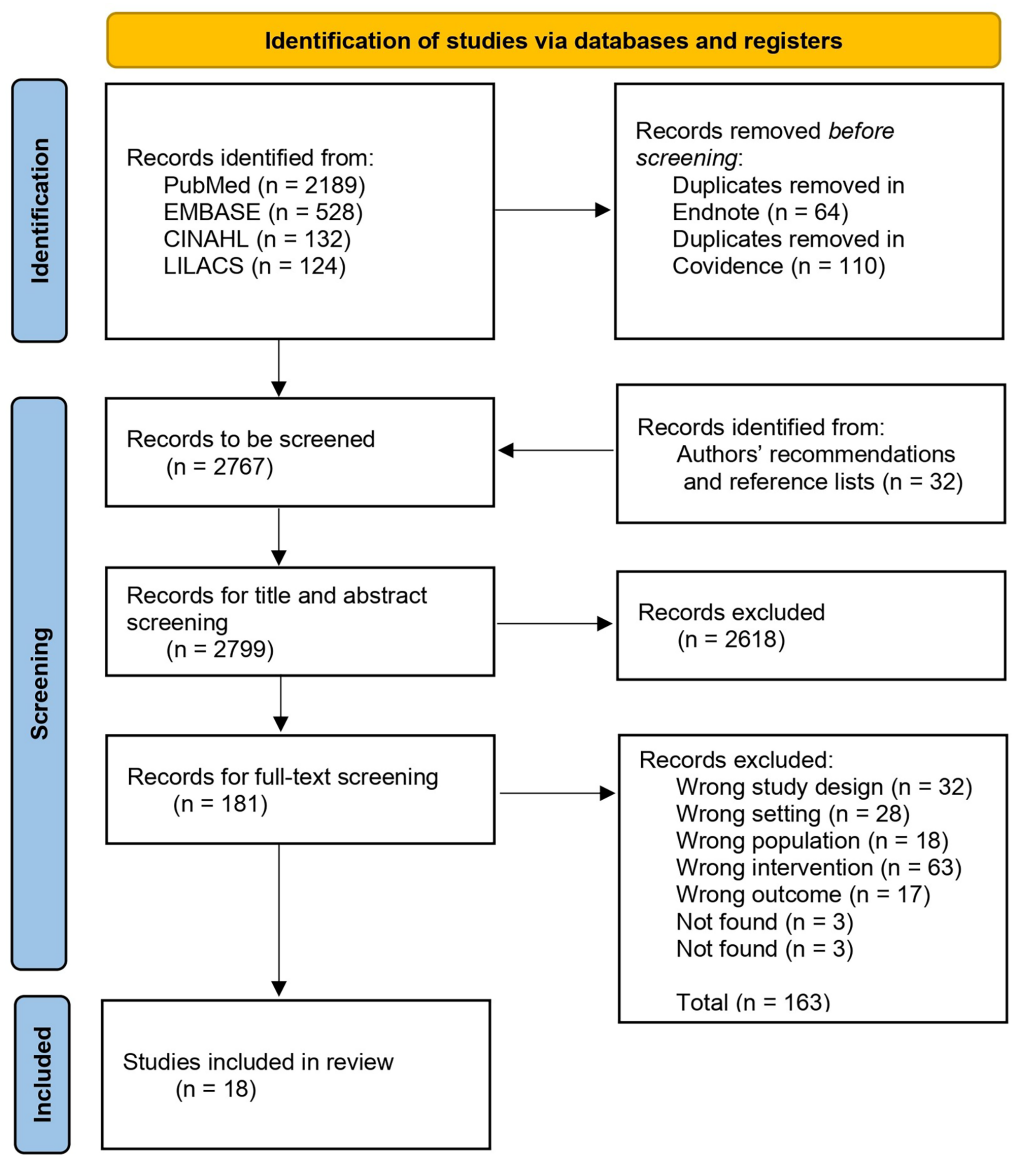
PRISMA flow diagram of the study selection process

The search strategy applied in 4 databases yielded 2973 articles, and after removing 174 duplicates and retrieving 32 records from authors’ recommendations and reference lists, 2799 articles were screened for title and abstract. From these, 181 full-text articles were assessed for eligibility, and a total of 18 articles met the inclusion criteria for the final review [18–35]. It should be noted that the review focused on the MFRs’ training simulating an out-of-hospital MCI context, thus studies were excluded from the review if the scenario was set in-hospital (e.g., ED room) [36–38] or in an ambulance [9]. The detailed selection process and reasons for exclusion are depicted in Fig. 1, and the summary of selected studies is presented in Table 3.

**Table 3:** Study characteristics (ordered from oldest year of publication to most recent)

1st Author (Year)	Country (Continent)	Study Design	MCI setting	Participants (number)	Intervention	Comparator	Training content	Training duration	Follow-up
Kizakevich (2007)	Iraq (AS)	Experimental (quasi)	Explosion	Emergency physicians (31)	VRS	NA	Triage, treatment, transport	2 days	No
Heinrichs (2008)	United States (NA)	Feasibility study	CBRNE	EMTs/Paramedics (8) (*scenario II*)	VRS (headset)	NA	Triage, decontamination, transport	NR	No
Vincent (2008)	United States (NA)	Experimental (pre-post)	Not described	Medical students (20)	VR (HMD and sensor gloves)	NA	Triage, treatment	16.82 min	No
Wilkerson (2008)	United States (NA)	Feasibility study	Explosion	Paramedics (15)	MR (human patient simulator)	NA	Triage, treatment	15-min orientation + 20 min (scenario)	No
Andreatta (2010)	United States (NA)	Experimental (RCT)	Explosion	Emergency medicine residents (15)	VR	C: Live simulation with Standardized Patient victims I: VR simulation	Triage	1 h lecture + scenario	After 2 weeks (post-test)
Knight (2010)	United Kingdom (EU)	Experimental (quasi)	Explosion	Emergency physicians & Emergency nurses & EMTs/Paramedics (91)	VRS	C: card-sorting exercise led by instructor I: VR simulation	Triage	15 min tutorial + 60 min scenario	No
Cone (2011)	United States (NA)	Experimental (quasi)	Car accident	Paramedic students (22)	VRS (joystick)	NA (same group: SALT vs. SMART triage systems)	Triage	1.5 h	After 3/6 months (10/23 students did post-test)
Cohen (2013)	United Kingdom (EU)	Feasibility study	Explosion	Emergency physicians (12) (*scenario I*)	VRS	NA	Triage, treatment	30 min	No
Farra (2013)	United States (NA)	Experimental (RCT)	Explosion & CBRNE	Nursing students (47)	VRS	C: web-based module I: web-based module + VR simulation	Triage, treatment, decontamination	30 min VR	After 2 months (41/47 students completed post-test)
Ingrassia (2015)	Italy (EU)	Experimental (crossover)	Car accident	Medical students (56)	VRS (joystick)	A: live simulation + VR B: VR + live simulation	Triage, treatment	2 h	No
Bajow (2016)	Saudi Arabia (AS)	Experimental (pre-post)	Building collapse & Fire	Medical students (29)	VRS (curriculum also included lectures, workshops, group discussions, and case studies)	NA	Triage, scene management	1 day	After 1.5 years (62% completed phone interview)
Foronda (2016)	United States (NA)	Experimental (pre-post)	Explosion	Nursing students (6)	VRS	NA	Triage, transport, communication	75 min.	No
Ngo (2016)	United States (NA)	Experimental (quasi)	Explosion & CBRNE & Earthquake	Emergency physicians (3), Emergency nurses (12), Emergency medicine residents (7)	VRS (curriculum also included lectures, tabletop exercises and high-fidelity hybrid simulations with mannequins and live actors)	NA	Triage, treatment	NR	No
Ferrandini Price (2018)	Spain (EU)	Experimental (quasi)	Not described	Emergency nursing students (67)	VR (helmet and smartphone)	C: Clinical Simulation with Actors I: VR simulation	Triage, decontamination, transport	NR	No
Follmann (2019)	Germany (EU)	Feasibility study	Explosion	Paramedics (31)	AR (SG)	C: without technical support **I1: AR with triage algorithm display** I2: AR with telemedical support	Triage	1.5 h	No
McCoy (2019)	United States (NA)	Feasibility study	Shooting	Emergency physicians (12), Emergency nurses (4), EMTs/Paramedics (5), Pharmacists (2) and other non-health related professions (8)	AR (MCI course via telesimulation: instructor wearing SG and also using live standardized patients and high-fidelity simulation mannequins)	NA	Triage	2.5 h	No
Mills (2020)	Australia (OC)	Experimental (crossover)	Shooting	Paramedic students (29)	VR (headset and controls)	A: Live simulation + VR B: VR + live simulation	Triage	2 days	No
Lowe (2020)	United States (NA)	Feasibility study	Shooting	Emergency physicians (96), Emergency medicine residents (66), medical students (13), EMTs/Paramedics (4), others (28)	VR (headset and controls)	NA	Triage, treatment (field intervention)	NR	No

Abbreviations – Continents: AS Asia, EU Europe, NA North America, OC Oceania; Study design: RCT randomized controlled trial; MCI settings: CBRNE Chemical, Biological, Radiological, Nuclear and Explosives; XR intervention: VR virtual reality, VRS – virtual reality simulation, MR mixed reality, AR augmented reality, HMD head-mounted display, SG smart glasses; Training duration: NR nor reported

While publication dates ranged from 2007 to 2020, half of the studies (*n* = 9) were published from 2015 onwards. Most studies (*n* = 10, 55%) were conducted in North America (particularly, United States), and the study designs included but were not limited to randomised controlled trials, pre-post and quasi-experimental studies, as well as feasibility studies. MCIs settings were varied, with explosion being the most commonly reported (*n* = 9), followed by CBRNE (*n* = 3) and shootings (*n* = 3). Participant populations were combinations of emergency physicians, emergency nurses, EMTs, paramedics and/or trainees (medical residents, nursing, paramedic or EMT students), and the number of participants per study ranged from 6 to 207. The total number of participants was 739 among all studies, trainees being the most frequent (*n* = 377), followed by emergency physicians (*n* = 154).

### Interventions, comparators and training features

The type of XR training used was primarily VR simulation (VRS) (*n* = 10) or VR (*n* = 5), with only two studies using AR and only one article using MR (Table 3). Examples of the technology used include the online virtual world Second Life as VRS [20, 22], VR system CAVE, which is a cave automatic virtual environment with stereo images projected on walls and floor [18, 35]. XR accessories such as head-mounted displays, sensor gloves or joysticks were utilized. Victims were played by actors, high-fidelity simulation mannequins, patient simulators or avatars. Of note, in all studies except one, participants were using the XR technology for themselves, however McCoy et al. described a MCI course that was taught via telesimulation, i.e., the instructor wore smart glasses to broadcast live through an interactive MCI scenario playing the role of a paramedic, evaluating each victim (live standardized patients and high-fidelity simulation mannequins) and verbalizing information needed for participants [31].

Seven studies had at least two training groups, five of which compared XR with other forms of training (e.g., card-sorting exercises, web-based modules, live simulation with actors or mannequins) and two were crossover studies where both groups were exposed to XR but changed the order of the intervention [27, 32]. The remaining studies had only one group that undertook either one or more training interventions. Among these, two studies used VRS as part of a curriculum that also included other teaching methods [19, 33], and one compared two triage systems using the same VRS method (SALT vs. SMART) [21].

Triage was taught in all included studies, either alone (*n* = 6) or in combination with other technical content such as treatment/intervention (*n* = 8), transport (*n* = 4), decontamination procedures (*n* = 3), scene management (*n* = 1), and even communication systems (*n* = 1). The duration of the training was not reported in all studies, but in those who did (*n* = 14), it ranged from 16 min to 2 days. Trainees were followed up only in 4 studies, from 2 weeks to 1.5 years after the training, mostly completing a post-test.

### Study outcomes and measurement tools

Table 4 includes a synthesis of the study outcomes and metrics. MCI training using XR was shown to be effective in 10 studies out of the 14 that did evaluate it (71.4%), and was mostly assessed as triage accuracy (*n* = 11) and time to triage (*n* = 8), along with knowledge acquired, treatment/intervention accuracy, and performance correctness. The experience of participants was reported in 13 studies (72.2%) and was described as positive in all of them. The measurement tools and metrics were varied across studies: using scores, checklists or ad hoc knowledge tests for effectiveness; and conducting focus groups, surveys with Likert scales, and interviews with open questions for participant experience. Two studies also assessed other physiological measures, such as heart rate or stress levels.

**Table 4:** Study outcomes: effectiveness of XR training and outcome measures (ascending ordered by Kirkpatrick level, and overall quality)

1st Author (year)	Outcomes		Measurement tools						Kirkpatrick level	Overall study quality
	Effectiveness	Participant perception	Triage accuracy	Time to triage	Treatment accuracy	Knowledge acquired	Performance correctness	Participant perception/experience		
Heinrichs (2008)	-	+*						Survey and focus group	I	Low
Ngo (2016)	-	+**						Likert scale (10-point)	I	Low
Cohen (2013)	-	+*						Likert scales (5-point) and interviews (semi-structured)	I	Medium
Kizakevich (2007)	-	+						Likert scale (5-point) and interviews	I	Medium
Knight (2010)	Yes	NR	Triage score as counts	Triage time			Performance score (triage step accuracy)		II	Medium
Andreatta (2010)	ND	NR	Triage score	Triage time		Pre/post knowledge test	Performance rating scale (scene management)		II	High
Cone (2011)	Yes	NR	Triage score	Triage time					II	High
Ingrassia (2015)	Yes	NR	Triage score as percentage	Triage time	Treatment score as percentage				II	High
Ferrandini Price (2018) §	ND	NR	Triage score						II	High
Foronda (2016)	ND	+				Pre/post knowledge test		Focus group (open questions)	I, II	Medium
Follmann (2019)	Yes	+	Triage score as percentage	Triage time				Questionnaire	I, II	Medium
Wilkerson (2008)	ND	+	Triage checklist	Triage time (in checklist)			Performance checklist (scene management)	Interviews (structured)	I, II	High
Farra (2013)	Yes	+				Pre/post knowledge test		Course evaluations	I, II	High
Mills (2020) §	Yes	+	Triage score as counts/ checklist	Triage time				Focus groups (open questions)	I, II	High
McCoy (2019)	Yes	+						Likert scale and survey	I, III	Medium
Vincent (2008)	Yes	+	Triage score	Triage time	Intervention score			Self-efficacy questionnaire (5-point Likert) and learner satisfaction (7-point Likert)	I, II, III	Medium
Bajow (2016)	Yes**	+**				Pre/post knowledge test		Likert scale (5-point) and open questions (course evaluations)	I, II, III	Medium
Lowe (2020)	Yes	+	Triage score		Intervention score			Likert scale (5-point)	I, II, III	Medium

Abbreviations – Effectiveness: ND Not determined, not enough information to determine. Participant experience: + positive, – negative, NR not reported

*Aggregated results of prehospital and in-hospital scenarios

**Aggregated results of XR with other training methods (not XR alone)

§ Mills also collected heart rate data (via smart watches and chest straps). Ferrandini Price also captured stress levels (measuring the activity of salivary α-amylase), heart rate, systolic and diastolic arterial pressure

Participants’ perceptions and experiences are summarized in Table 5. In general, trainees showed strong interest and high acceptability of the XR training, pointing out how engaging, enjoyable and fun it was. Some people also expressed the usefulness, relevance, and added educational value of this experience that provided them with a better understanding. Immersive and realistic scenarios increased the intensity of the activity, along with auditory cues such as background sounds, which also raised their anxiety levels. Furthermore, participants appreciated the immediate feedback of the XR system, as well as the possibility to repeatedly practise in a variety of scenarios that are difficult to train in real life. One study highlighted the inability to replicate the human emotion in the VR scenario to the same extent as live simulation [32]. Participants from two studies also suggested improvements, such as having repercussions to their inappropriate interventions [25], and accounting for bystander and crowd control in the scenario [35]. Though rare, some participants felt initial motion sickness [35] and had difficulties maneuvering the controls [22].

Regarding the Kirkpatrick evaluation model, 14 studies reported participants’ feelings about the training program (level I) and/or the level of expertise achieved (level II) (77.8% each). Four studies (22.2%) assessed the self-efficacy or transfer of training (level III), but no study was classified as level IV. Only three studies reported all 3 levels: reaction, learning and behaviour.

### Risk of bias

The relevancy, reliability, validity, and applicability of all included studies were appraised. As a result, overall study quality was classified as high in 7 studies, medium in 9, and low in 3. Ethics procedures were not described in 6 studies. While some studies accounted for potential confounders, nine studies did not properly address possible bias in the methodology, e.g., trainees with varying levels of prior knowledge might influence the overall training effect. One study did not provide the participant number by occupation [29], and four studies did not report clear findings specific to the population of interest, but rather presented results of the MFRs in combination with in-hospital scenarios [20, 26] or other training methods [19, 33]. A detailed description of the risk of bias assessment is available as Additional file 1.

**Table 5:** Participants’ perceptions and experiences reported (order by XR intervention and Kirkpatrick level)

Author (Year)	XR	Kirkpatrick level	Perceptions from participants
Kizakevich (2007)	VRS	I	Participants provided positive comments regarding the course elements, including: didactic course & presenter, simulation realism & navigation; simulation content & responsiveness; and simulation learning content.
Heinrichs (2008)	VRS	I	Participants thought that game-based training was as or more effective than traditional methods (62%), and they reported that the game environment is useful for initial training (56%) and for refresher training (75%).
Cohen (2013)	VRS	I	MFRs highlighted that VRS enables training in environments that are difficult/impossible to train in real life. Participants agreed that the scenarios were realistic visually and clinically.
Ngo (2016)	VRS	I	Participants found the scenarios to be realistic, educational, and relevant to their practice, and believed that they gained valuable knowledge and skills to prepare for future disasters
Farra (2013)	VRS	I, II	Participants gave positive comments about VRS (80%), regarding the visuals, the realistic experiences, and a better understanding of zones of triage. Some participants had difficulty navigating the VRS due to difficulties in maneuvering the controls and operating of teleports. Only one of the participants provided negative feedback reporting that VRS was not realistic.
Foronda (2016)	VRS	I, II	Participants emphasized it was a “fun way to learn”, and appreciated the feature of immediate feedback. They preferred this learning strategy over reading, while they suggested improvements, such as having repercussions to their inappropriate interventions.
Bajow (2016)	VRS	I, II, III	Most participants found the course interesting (76%), and gave positive comments about the instructors (93%). They stated that the course was appropriate and relevant to their medical education, and expressed strong interest in VRS. When following up with students 1.5 years later, 62% of them were reached out and reported to have changed their attitude and being less stressed/more confident.
Mills (2020)	VR	I, II	VR experience was graphically realistic and comparable to the live simulation with respect to visual and auditory information provision. In VR, they could better focus on their triage skills but VR was unable to replicate the human interaction and emotional immersion element of the experience to the same extent as live simulation.
Vincent (2008)	VR	I, II, III	Participants’ self-efficacy increased over time: they became more confident in prioritizing treatment and resources, as well as identifying high-risk patients, thus being able to learn how to be an effective first responder.
Lowe (2020)	VR	I, II, III	Participants found the VR experience engaging (median = 5) and enjoyable (median = 5), and they would like to see VR integrated in medical education, specifically for disaster training and pediatric training. Most felt that VR was more immersive than mannequin-based simulation training (median = 5). They felt more prepared for adolescent MCIs.
Wilkerson (2008)	MR	I, II	The simulation realism raised the anxiety levels, which increased the intensity of the experience. Background noise, chaos, and radio traffic (two-way interactions) contributed to the reality of the simulation. This technology allowed participants to consolidate correct responses by repeatedly running through variations of the scenario. Weaknesses: some participants experienced initial motion sickness, and the scenario did not account for bystander and crowd control.
Follmann (2019)	AR	I, II	The Smart Glasses showed sufficient acceptance among subjects, with 73% (8/11) participants reporting good or very good usability. Most subjects confirmed compatibility with the personal protective equipment in the event of a disaster.
McCoy (2019)	AR	I, III	Participants provided a favorable response regarding their thoughts, feelings, and attitudes toward learning EMS-based content via telesimulation. Particularly, they reported that virtual simulation was more effective to learn the MCI triage method and added educational value beyond learning from standard lectures (e.g., tabletop exercises). Participants also found wearable technology to be an effective tool to transmit critical patient information between providers in the prehospital setting. Additionally, participants reported that the telesimulation course enhanced their ability to provide care for patients involved in a MCI.

## Discussion

XR technology has been introduced in recent years as a new approach for disaster medicine education [7, 10]. A systematic review of the scientific literature was conducted to assess the effectiveness and MFRs’ experience regarding XR training for MCI, including 18 studies. The synthesis of data indicated that XR was an effective tool for prehospital MCI training by means of improved triage accuracy, triage time, treatment accuracy, performance correctness and/or knowledge acquired. These XR systems were well perceived by MFRs, who expressed their interest and satisfaction towards this learning experience and emphasized its usefulness and relevance. Accordingly, XR seems to be a suitable and promising way to consolidate and gain valuable knowledge and skills to prepare for future disasters.

Managing these high-severity, low-frequency events present significant challenges and requires high-level performance, yet MFRs have little opportunity to rehearse and improve disaster response. One of the most essential advantages of XR is the possibility of recreating complex and inaccessible scenarios to train safely [20], as well as the opportunity to recurrently practise putting their knowledge into action [35]. Therefore, this technology could enhance knowledge capture and reduce the costs in training [32] due to the versatility and flexibility to create adaptative environments [12]. Moreover, during MCIs, emotional distress could adversely affect health professionals’ performance and may impact the speed and accuracy at which patients are assessed and triaged [3, 18, 39]. The capability to manage uncertainty and surprising situations is crucial [40], and in that sense, realistic simulations and repetition could allow MFRs to improve not only their skills but their confidence and self-perception too. Furthermore, an additional benefit of XR technology over traditional training methods is the ability to provide individual feedback to participants, either immediate or after the session, which is necessary for learning and retention. Some of the XR systems used were able to record the scenario from the point of view of each participant, thus enabling accurate and structured debriefing, otherwise difficult in large-scale simulation exercises [25, 35].

Virtual worlds and immersive XR have the potential to influence student engagement and motivation, as well as skill acquisition and learning. Similarly to our colleagues [12], our findings indicated there is no unique way to determine and measure the achieved knowledge or abilities: the diversity found in outcomes and measurement tools, and the lack of use of validated scales hindered comparability of results. While some studies were not able to demonstrate the effectiveness of the XR in terms of triage accuracy or performance correctness among other outcomes (Kirkpatrick level II), MFRs did express very positive feelings and opinions towards the technology used, and even enhanced self-efficacy, which is paramount for disaster response competency [41]. Therefore, special importance should be given to the participants’ perceptions and experiences when assessing the impact of the training, to also evaluate their reactions and how well behaviours learned are performed on the job (Kirkpatrick levels I and III).

Simulation is a key enabling technique — not technology— for improving patient safety [42]. Summarized below are recommendations and suggestions to take into consideration when designing, implementing or upgrading an MCI course with XR simulation:

- MCI training
  - In regards to the training content, most studies focused on first triage, whereas disaster response goes beyond the correct classification of victims; it also entails other tasks, including scene management (identifying and zoning high-risk areas), assessment of immediate needs, protection and safety procedures, or calling for additional resources [18, 35, 43]. Emphasis should be placed in comprehensive learning contents for disaster education.
  - The included studies mainly addressed technical skills (e.g., triage, treatment/ intervention). These studies found XR especially useful for training the making of decisions rather than teaching practical skills, such as opening an airway or control a hemorrhage [23]. Nonetheless, this novel technology also has the potential to enhance non-technical skills, including leadership and communication [20]. Further core competencies for disaster response, such as coordination or hazard perception, could also be developed using this environment [44].
  - While multi-agency response is vital in MCIs, only 4 of the 18 studies combined different healthcare professionals such as nurses, EMTs, and physicians. Other first responders such as police officers or firefighters could also be included as part of the training exercises [18], which would be highly important for teamwork, cooperation and communication [45].
  - When planning a disaster course curriculum, after the educational needs are identified and a learning strategy is decided, the evaluation of results should be defined [46, 47]. For this purpose, it is strongly encouraged to plan the evaluation keeping in mind the different levels of Kirkpatrick’s model [16]and to also aim at measuring the impact of the training on health outcomes (level IV). In these assessments, it would also be recommended to use validated measurement tools (when available) for comparability.
- XR realism
  - Distress plays a major role in learners’ performance, thus special attention should be given to their stress levels [3]. Background radio noises and other stressors were reported to amplify the intensity of the experience [32]. Accordingly, other physiological measures such as stress levels and heart rate should be considered, as assessed in two studies [23, 32]. As well, audio-visual signals and crowds of bystanders could be incorporated to enhance the realism of the scenario [35], and even other sensory inputs (e.g., wind or gas smell) could be introduced.
  - While it was only reported in one study, interviewed participants felt initial motion sickness from using the VR headsets [35]. Participants’ fatigue when immersing themselves in the virtual environment should be assessed. As such, future studies should look into this issue, and should consider the appraisal of the 5 fatigue domains (general, visual, social, motivational and emotional) [48].
  - Lastly, while avatars and patient simulators used in XR can be notably realistic, some participants reported that these were unable to replicate human interaction and emotion to the same extent as a live simulation with actors [32]. Further research should aim to address how to better depict the humanity of and the interaction with the victims.

### Strengths and limitations

Among the strengths of this review are the in-depth search strategy, the specific eligibility criteria, and the use of a validated quality appraisal tool (i.e., MetaQAT). As a result, this research provides a comprehensive overview of the current literature available regarding innovative training methods for disaster preparedness that could be of interest for MFRs, and especially for policymakers, emergency medical service agencies, and disaster management authorities.

Despite the methodological rigor applied in this review, this study presents several limitations. Firstly, the number of participants in most of the studies was limited, which might hinder the statistical significance of results. As well, most of the studies did not follow-up with participants and thus, did not assess knowledge retention. Accordingly, further research should focus on testing or implementing XR training at a larger scale and should also reach out to learners after the training for: (a) inquiring how well the acquired knowledge is maintained in the long term, and (b) measuring whether behaviours/performance are applied on the job. Secondly, some studies reported aggregated results with other participants or training methods, thus it was not possible to adequately infer the effectiveness and perception of the target population. Lastly, studies were not immune to bias. For instance, one study reported that the VRS facilitator was an advocate for this technology, and it is unknown how it could have swayed students’ responses [25]. Therefore, it is advised to better address possible biases or confounders when planning the research methodology and analyzing the data, and to clearly outline the study findings.

## Conclusion

This systematic review described the current state of knowledge regarding the applications of XR for prehospital emergency training in MCIs. This research offers supportive evidence of the usefulness and significance of this novel technology provided with flexible environments that allow users to enhance their skills and confidence when facing forthcoming disasters. The findings of this research summarize recommendations and suggestions for the implementation, upgrade and/or assessment of this teaching method, thus providing a direction for future researchers seeking to streamline the education of MFRs.

### Electronic supplementary material

Below is the link to the electronic supplementary material.

**Additional file 1.** Summary of the risk of bias assessment using MetaQAT

## Data Availability

No datasets were generated or analysed during the current study.

## Abbreviations

MCI: Mass casualty incident
MFR: Medical first responder
XR: Extended reality
VR: Virtual reality
AR: Augmented reality
MR: Mixed reality
CBRNE: Chemical, Biological, Radiological, Nuclear and Explosives
EMT: Emergency medical technician
