# Supplementary figures and images for: Extended reality training for mass casualty incidents: a systematic review on effectiveness and experience of medical first responders
Source: Int J Emerg Med. 2024 Aug 23;17:99. doi: 10.1186/s12245-024-00685-3 (PMC11342566; doi:10.1186/s12245-024-00685-3)

**Additional file 1.** Summary of the risk of bias assessment using MetaQAT


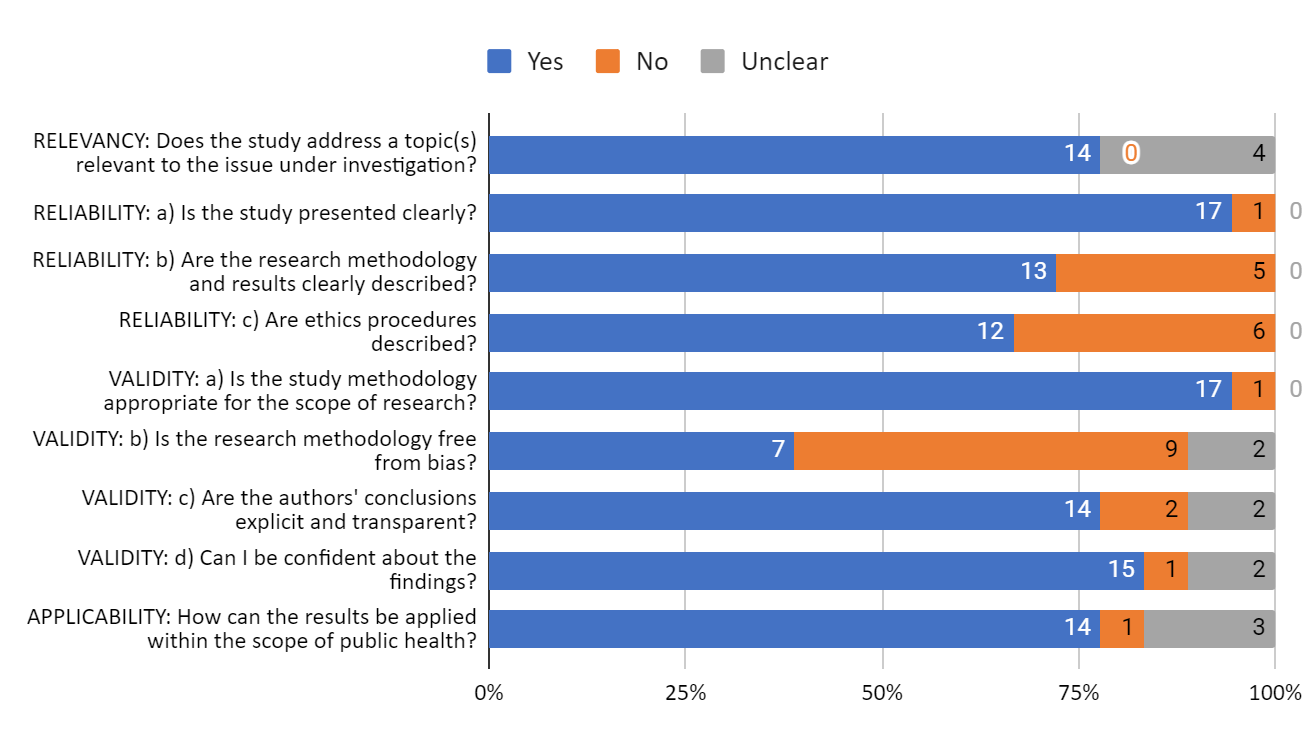

Supplement: Supplementary file 1 — Additional file 1. Summary of the risk of bias assessment using MetaQAT [file 12245_2024_685_MOESM1_ESM.docx]
